# Multiple Routes to Color Convergence in a Radiation of Neotropical Poison Frogs

**DOI:** 10.1093/sysbio/syad051

**Published:** 2023-08-10

**Authors:** Evan Twomey, Paulo Melo-Sampaio, Lisa M Schulte, Franky Bossuyt, Jason L Brown, Santiago Castroviejo-Fisher

**Affiliations:** Department of Wildlife/Zoo Animal Biology and Systematics, Faculty of Biological Sciences, Goethe University Frankfurt, Max-von-Laue-Str. 13, Frankfurt am Main 60438, Germany; Departamento de Vertebrados, Museu Nacional, Universidade Federal do Rio de Janeiro, R. Gen. Herculano Gomes 41, Rio de Janeiro 20941-360, Brazil; Department of Wildlife/Zoo Animal Biology and Systematics, Faculty of Biological Sciences, Goethe University Frankfurt, Max-von-Laue-Str. 13, Frankfurt am Main 60438, Germany; Amphibian Evolution Laboratory, Biology Department, Vrije Universiteit Brussel, Pleinlaan 2, Brussels 1050, Belgium; School of Biological Sciences, Southern Illinois University, 125 Lincoln Dr., Carbondale, IL 62901, USA; Departamento de Zoología, Universidad de Sevilla, Av. de la Reina Mercedes, Seville 41012, Spain

**Keywords:** Carotenoids, coloration, convergent evolution, Dendrobatidae, pterins, ultraconserved elements

## Abstract

Convergent evolution is defined as the independent evolution of similar phenotypes in different lineages. Its existence underscores the importance of external selection pressures in evolutionary history, revealing how functionally similar adaptations can evolve in response to persistent ecological challenges through a diversity of evolutionary routes. However, many examples of convergence, particularly among closely related species, involve parallel changes in the same genes or developmental pathways, raising the possibility that homology at deeper mechanistic levels is an important facilitator of phenotypic convergence. Using the genus *Ranitomeya*, a young, color-diverse radiation of Neotropical poison frogs, we set out to 1) provide a phylogenetic framework for this group, 2) leverage this framework to determine if color phenotypes are convergent, and 3) to characterize the underlying coloration mechanisms to test whether color convergence occurred through the same or different physical mechanisms. We generated a phylogeny for *Ranitomeya* using ultraconserved elements and investigated the physical mechanisms underlying bright coloration, focusing on skin pigments. Using phylogenetic comparative methods, we identified several instances of color convergence, involving several gains and losses of carotenoid and pterin pigments. We also found a compelling example of nonparallel convergence, where, in one lineage, red coloration evolved through the red pterin pigment drosopterin, and in another lineage through red ketocarotenoids. Additionally, in another lineage, “reddish” coloration evolved predominantly through structural color mechanisms. Our study demonstrates that, even within a radiation of closely related species, convergent evolution can occur through both parallel and nonparallel mechanisms, challenging the assumption that similar phenotypes among close relatives evolve through the same mechanisms.

The independent evolution of similar phenotypes in different lineages—convergent evolution—is a striking and ubiquitous feature of life (McGhee 2011). Well-known examples of convergent evolution include image-forming eyes in dinoflagellates, cephalopods, and vertebrates (Leander 2008), repeated pelvic reduction in sticklebacks (Bell et al. 1993), and recurrent ecomorph evolution in *Anolis* lizards (Losos et al. 1998). Because convergent evolution indicates that certain phenotypes evolve repeatedly and predictably, its existence supports the view that “replaying life’s tape” would result in similar, deterministic outcomes due to external forces such as natural selection. The best examples supporting this view are those of complex and clearly adaptive traits evolving across deep timescales (Powell and Mariscal 2015), which minimizes the extent to which homologous mechanisms (e.g., conserved genes, pathways) can explain the convergence (Leander 2008).

On the other hand, because closely related species share more homologous traits than distantly related species, one expects (and frequently observes) that convergence among close relatives is due to homologous underlying mechanisms (frequently termed “parallel evolution”) (Shapiro et al. 2006; Rosenblum et al. 2010; Conte et al. 2012). Such cases support the view that internal forces (e.g., homology) are important facilitators of recurrent phenotypic evolution (Pearce 2012). However, there are a handful of examples of “nonparallel” convergence (i.e., convergence via different mechanisms, sensu Rosenblum et al. 2014) among close relatives, as well as parallel convergence among distant relatives (e.g., shared bone structure in tetrapod wings) demonstrating that convergence can occur through parallel or nonparallel pathways regardless of phylogenetic depth (Calboli et al. 2003; Wilkens and Strecker 2003; Leander 2008; Steiner et al. 2009; Kemppainen et al. 2021). As with distant relatives, nonparallel convergence among close relatives better demonstrates the importance of external selection pressures in that homologous mechanisms are not a prerequisite for convergent phenotypes to evolve. Thus, the extent to which convergence can inform us about evolution is in large part dependent on our understanding of the mechanistic basis of convergent phenotypes and determining whether convergence among close relatives occurs through similar or different mechanisms.

It is important to clarify two points when discussing parallel versus nonparallel evolution. The first is that phenotypes have hierarchical levels of causality (e.g., nucleotide, gene, pathway, physical mechanism), so convergence can occur through similarity at different causal levels. Independent changes to the same gene (or even the same nucleotide) can yield identical phenotypic effects; similarly, different genes can be co-opted for the same function (Elmer and Meyer 2011), as can different physical mechanisms (e.g., pigmentary vs. structural coloration, discussed below). Second, although phenotypic convergence is homoplasy, some of the causative mechanisms that underlie these phenotypes may be homologous. For example, mandibular (“true”) teeth were lost in the ancestor to frogs over 200 million years ago, and regained in a single frog species, *Gastrotheca guentheri* (Boulenger 1882; Wiens 2011). Given their anatomical similarity to other amphibian teeth, this suggests that true teeth in frogs arose from an ancient developmental pathway shared among all amphibians, perhaps through the deactivation of a suppressor regulatory gene (Paluh et al. 2021). In this example, it is easy to see how a conserved pathway for tooth development may have facilitated the re-evolution of this homoplasic trait.

Animal coloration has played a prominent role in the study of convergence (Bates 1862; Hoekstra et al. 2006; Rosenblum et al. 2010; Friedman et al. 2014; Kratochwil et al. 2018). Bright, (i.e., non-melanic) coloration is produced from a wide variety of physical mechanisms, meaning that ample opportunity exists for convergence via different evolutionary paths. For example, blue coloration in animals is usually structural (but see Goda and Fujii 1995; Rüdiger et al. 1968; Taboada et al. 2020; Yu et al. 2008 for examples of blue pigments). However, a wide variety of blue-producing structures have evolved, including guanine-based iridophores (fish, reptiles, and amphibians; Bagnara et al. 2007), reflectin-based iridophores (cephalopods; Crookes et al. 2004), collagen arrays (bird and mammal skin; Prum and Torres 2003, 2004), the spongy medullary layer of avian feather barb rami (Prum 2006), and beta-keratin/melanin/air matrices in avian feather barbules (Prum 2006). Other colors, such as yellow, orange, and red, are more commonly produced from pigments such as carotenoids and pterins. However, structural color can also play an important or even predominant role in generating these colors (Morrison 1995; Teyssier et al. 2015). Overall, convergent color evolution is not only common, but can occur through parallel and nonparallel mechanisms.

Here, we study the physical mechanisms underlying a color radiation in the Neotropical poison frog genus *Ranitomeya* (family Dendrobatidae) in a comparative phylogenetic framework. This is a relatively young group (~10 million years; Santos et al. 2009) consisting of 16 species, and exhibits extreme color variation, spanning the visible spectrum from blue to red (Brown et al. 2011). Dendrobatids, like many other amphibians, produce color through a stacked arrangement of dermal cells often referred to as the “dermal chromatophore unit” (Bagnara et al. 1968; Kuriyama et al. 2006; Twomey et al. 2020b). The most superficial layer of the dermal chromatophore unit are the xanthophores/erythrophores, which contain brightly colored pigments such as carotenoids and pterins (Obika and Bagnara 1964; Bagnara 1966). These two classes of pigments originate from unrelated pathways, being of dietary and endogenous origin, respectively (Giordano et al. 2003; Ziegler 2003; Toews et al. 2017). Below are iridophores, which contain stacks of refractive guanine crystals interspersed with cytoplasm that produce structural coloration (Bagnara et al. 2007). Melanophores are the deepest cell type, containing the black pigment melanin. Besides producing black coloration, melanin contributes to structural color by absorbing incoherently scattered light (Shawkey and Hill 2006). Importantly, due to the broad overlap in terms of colors produced by each mechanism, this means that similar colors in different species can be produced by various combinations of carotenoids, pterins, and iridophores, or by each mechanism alone (Teyssier et al. 2015; Twomey et al. 2020b; Stuart-Fox et al. 2021a).

Specifically, we ask the following question: Does color convergence among closely related species occur through the same or different mechanisms? To address this, we provided a phylogenetic framework for this group, including all 16 nominal species, using ultraconserved elements. Within this framework, we analyzed the evolution of bright dorsal coloration to examine whether similar phenotypes are convergent. Finally, we analyzed skin pigments and spectral reflectance data to characterize the underlying mechanisms of color convergence, allowing us to identify common or independent evolutionary causes of the same observed phenotypes.

## Materials and Methods

### Sample Collection and Data Use

We aimed to sample the color diversity in *Ranitomeya* as completely as possible across nominal species and color morphs of polymorphic species. To do so, we sampled *Ranitomeya* populations throughout Peru and western Brazil. During our sampling in western Brazil, we also discovered several *Ranitomeya* populations with unique coloration but of unclear taxonomic status due to unresolved species limits. For this reason, the terminals in our phylogeny have no consistent taxonomic rank and are herein referred to as taxa.

We collected pigment and reflectance data from 11 *Ranitomeya* taxa throughout the Amazonian regions of eastern Peru and western Brazil. For an additional 10 taxa, we took pigment and reflectance data from previous studies on color mechanisms in poison frogs (Twomey et al. 2020a, 2020b). Supplementary Table S1 provides information on sampling localities and sample sizes for the pigment and reflectance data sets. For the metrics used in the phylogenetic comparative analyses (dorsal color metric PC1, reflectance λ_max_, absorbance-based pigment concentrations), we calculated each metric on a per-individual basis and then averaged at the level of taxon. Of the 38 samples included in the phylogenetic analysis, 15 were from Guillory et al. (2019), while the remaining 23 are new to this study. See Supplementary Table S2 for overview of sampling localities and accession numbers for samples in the phylogenetic analysis.

### Reflectance Spectrometry and Quantification of Dorsal Color

**Figure 1.**
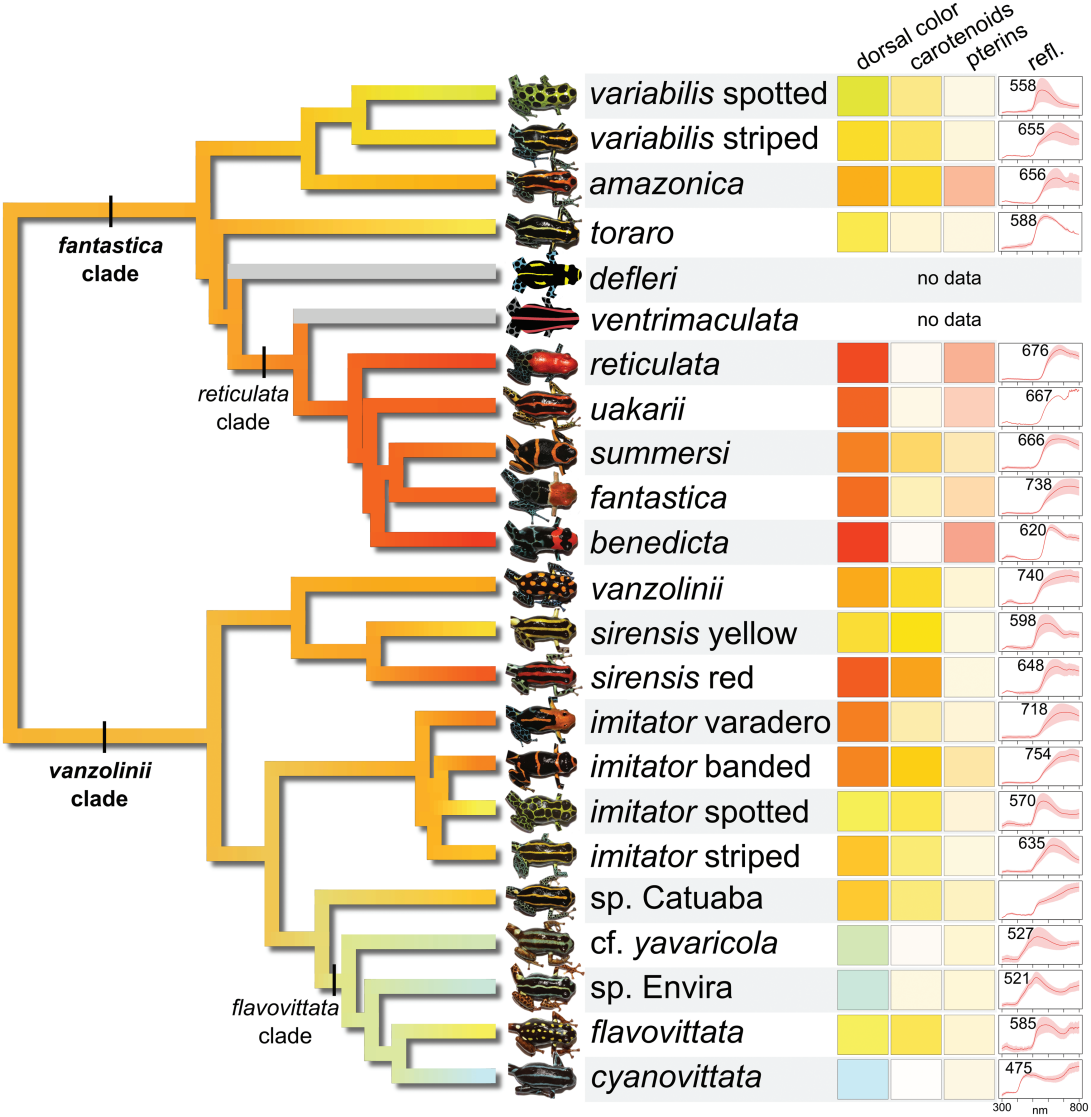
Color evolution in *Ranitomeya.* On the left, a maximum-likelihood ultrametric phylogeny of *Ranitomeya* based on 1550 ultraconserved element loci. Branches are colored according to ancestral state reconstructions of dorsal coloration. On the right, boxes show dorsal color, pigment colors, and reflectance spectra. Dorsal color: Red-Green-Blue (RGB) color measured from dorsal photographs (i.e., the observed frog color that was mapped onto the phylogeny). Carotenoids: RGB rendering of the chloroform extract, modeled onto a white reflector. Pterins: RGB rendering of the ammonia extract, modeled onto a white reflector. Refl: Dorsal reflectance spectrum; numbers indicate the wavelength of peak reflectance (reflectance λ_max_). RGB renderings, reflectance spectra, and reflectance λ_max_ values represent population averages; in the case of reflectance spectra the solid red line is the mean and red shaded area is the standard deviation. RGB renderings of pigment absorbance spectra were done with the spec2rgb function of pavo2 (see Supplementary Material for more details). For complete phylogeny with outgroups see Supplementary Figure S1.

Reflectance spectrometry was done with a Stellarnet Silver-Nova spectrometer (190–1100 nm range) and Spectrawiz software version 5.33. Reflectance measurements were taken on each frog at 4 points on the brightly colored parts of the dorsum (right/left head and right/left mid-back, excluding black regions) and averaged to a single reflectance spectrum, except for frogs whose head and body were a different color (i.e., *R. fantastica* and *R. benedicta*, Fig. 1), in which case only the two head measurements were averaged. Reflectance measurements were taken relative to a halon white reflectance standard, with illumination and read angle at 45° relative to the skin, at a fixed distance of 3 mm using a custom 3D-printed probe tip. UV-VIS and VIS-NIR measurements were taken separately, using a Stellarnet SL3 deuterium lamp for the former and a Stellarnet SL1 halogen lamp for the latter. As the two readings overlapped from 400 to 700 nm, it was possible to splice the two spectra to a single spectrum with a clean signal from approximately 220–1100 nm. Reflectance spectra were plotted and analyzed in R version 3.6.3 (R Core Team 2020) using the Pavo2 package (Maia et al. 2019).

As the wavelength of peak reflectance (λ_max_, commonly referred to as hue or spectral location) has been shown to be correlated to the thickness of iridophore platelets in poison frogs and other organisms (Morrison 1995; Twomey et al. 2020b), we used λ_max_ of the reflectance spectrum as a proxy for structural color hue and included this trait in our comparative analysis.

To investigate dorsal color evolution in *Ranitomeya*, we used two methods. First, for visualization purposes, we measured RGB values from dorsal photographs and mapped the individual color channels onto the phylogeny as shown on Figure 1 (additional details are given in the Supplementary Material). For a more rigorous analysis of dorsal color, we also derived a dorsal color metric from the spectral reflectance data. For this analysis, the main challenge was to reduce the dimensionality of the spectral reflectance data to one or two meaningful color metrics that could be analyzed. To do this, we used the vismodel and colspace functions of Pavo2 to calculate quantum catch data for each of 4 cone types in a tetrachromat visual system, reducing the dimensionality to 4 columns per individual (i.e., quantum catch values for u, s, m, and l cones). For each individual, these calculations were done separately on each dorsal reflectance measurement, and quantum catches were averaged per cone type per individual. Vismodel was calculated with the following parameters: visual = “avg.uv,” achromatic = “bt.dc,” relative = TRUE, illum = “ideal). This visual system was chosen as it represents an average avian UV visual system, and is thus representative of an ecologically important viewer (Maan and Cummings 2012). To further reduce the dimensionality of the quantum catch data, we performed a principal components analysis using the prcomp function in R, centering and scaling the data. We retained the first two principal components for further analysis.

### Pigment Analyses

We analyzed skin pigments (carotenoids and pterins) using absorbance spectrometry (Supplementary Fig. S1) and thin-layer chromatography (TLC), following Twomey et al. (2020a, 2020b). These approaches yield complementary data sets with different strengths and weaknesses. Absorbance spectrometry of bulk pigment extracts gives a good measure of overall pigment concentrations and their color, as the processing of extracts is absolutely minimized; however, it provides limited information on pigment identity. TLC, on the other hand, allows for identifications of specific pigments but is far more labor intensive and involves much more sample processing, potentially leading to a loss of quantitative information. Therefore, for the purposes of this study, quantitative pigment information was taken from absorbance spectra while TLC was used only for qualitative determination of pigment presence/absence. Details on TLC of carotenoids and pterins are given in the Supplementary Material. We identified individual carotenoid and pterin pigments following the methods described in Twomey et al. (2020a, 2020b). Absorbance spectra of carotenoid and pterin extracts were plotted with Pavo2 (Maia et al. 2019). Carotenoid and pterin concentrations were determined from absorbance spectra using the Beer-Lambert law (Hudon and Brush 1992) as detailed in the Supplementary Material.

Our pigment analyses resulted in two quantitative traits derived from absorbance spectra (carotenoid concentration at λ_max_ and pterin concentration at 500 nm, which corresponds to the drosopterin absorbance peak), and 7 discrete traits (5 carotenoids and two pterins) representing all skin pigments with a color in the visible range (presence/absence of the carotenoids beta-carotene, lutein, C-4 ketocarotenoids, canary xanthophyll, eschscholtzxanthin-like, and the pterins drosopterin and sepiapterin) derived from the TLC results. These were included in our phylogenetic comparative analysis.

### Phylogenetics

We sampled tissues from as many species and color morphs as possible in *Ranitomeya* to infer a phylogeny for use in our comparative analysis of color evolution. We used ultraconserved elements (UCEs), which are genome-wide molecular markers containing an “ultraconserved” core region with increasingly variable flanking regions, facilitating the identification and alignment of homologous sequences (Faircloth et al. 2012). Such markers have been used to resolve phylogenies at both deep and shallow timescales, including dendrobatid poison frogs (Crawford et al. 2012; Smith et al. 2013; Muell et al. 2022). We were able to include all 16 currently recognized species of *Ranitomeya* in the phylogeny, including *R. defleri* and *R. ventrimaculata* (for which we had no pigment or color data), plus two potentially undescribed species (Fig. 1, “sp. Catuaba” and “sp. Envira”). Three species of *Ranitomeya* were represented by multiple color morphs (*R. variabilis, R. sirensis*, and *R. imitator*; Fig. 1), resulting in a total of 23 terminals in our phylogenetic ingroup. Given the potentially strong effect of outgroup sampling on ingroup topology (Grant 2019), we included many outgroup samples from the subfamily Dendrobatinae, with at least one species per genus, except for *“Colostethus” ruthveni* (Supplementary Fig. S2).

Genomic DNA was extracted from Brazilian tissue samples with the Promega Wizard extraction kit and from Peruvian tissue samples with the Qiagen DNeasy extraction kit. We sent extracted DNA to RAPiD Genomics (Gainesville, FL, USA), where library preparation and Illumina sequencing of UCEs was done following Faircloth et al. (2012). The Tetrapod UCE 5Kv1 probe set was used to target 5060 UCE loci. Roughly 1–2 million paired-end 150 bp reads were sequenced per sample.

We used Phyluce 1.6 (Faircloth 2016) to clean sequencing reads, assemble reads into contigs, and extract and align UCE loci for phylogenetic analyses as detailed in the Supplementary Material. We also used Phyluce to create a 95% complete data matrix, that is, one where only loci present in 95% or more of taxa were retained.

Aligned loci were concatenated into a single alignment for phylogenetic analysis. We searched for the best partition scheme and model of nucleotide evolution using ModelFinder (Kalyaanamoorthy et al. 2017), with the commands *-m testnewmergeonly* and *-rcluster 30* and the Bayesian information criterion (Schwarz 1978). We performed 200 independent tree searches under the maximum likelihood (ML) criterion in IQ-TREE 2.0.6 (Minh et al. 2020b), with the command *-nstop* set to 500, the option *-allnni* activated, and using the models and partitions resulting from ModelFinder (Chernomor et al. 2016). We used default settings for other parameters.

We also calculated 1000 replicates of the ultrafast bootstrap approximation (UFBOOT) using IQ-TREE with default settings and the partitions selected by ModelFinder (Minh et al. 2013; Hoang et al. 2018). We used the SumTrees package in DendroPy v4.4.0 (Sukumaran and Holder 2010) to add UFBOOT frequencies to the best ML tree. To quantify genealogical concordance, we calculated gene and site concordance factors (gCF and sGF, respectively) using IQ-TREE (Minh et al. 2020a).

To assess phylogenetic resolution, we collapsed nodes incompatible with those from slightly suboptimal trees, defined as the trees from our 200 independent searches that differ from the best reported tree by < 0.1 log-likelihood unit. This value is typically an order of magnitude less than the change in log-likelihood after adding a single autapomorphy to the data matrix (Simmons and Kessenich 2020).

The UCE sequence alignment, partition scheme, and phylogenetic trees are available on Dryad at https://doi.org/10.5061/dryad.gf1vhhmsn

### Comparative Analyses

To examine how frog color and coloration mechanisms evolved within *Ranitomeya*, we used phylogenetic comparative methods implemented in the R package phytools version 0.6-99 (Revell 2012). We pruned the full tree (Supplementary Fig. S2) using the drop.tip function of phytools to remove terminals lacking color/pigment data (mostly outgroup taxa) and made our phylogeny ultrametric with an arbitrary calibration (total tree age = 1) using the chronos function of the R package ape version 0.54 (Paradis and Schliep 2019). We compared ultrametric phylogenies created with each of the 3 available models of substitution rate variation (correlated, relaxed, and discrete), two levels of the lambda parameter (0 and 1), and 1–5 rate categories for the discrete model (nb.rate.cat parameter). For further analyses, we selected the ultrametric tree with the lowest phylogenetic information criterion (PHIIC or ΦIC; Paradis 2013), which was the discrete model with a single rate, equating to a strict-clock. All subsequent analyses were based on this tree.

We explored the mode of trait evolution by fitting Brownian motion (BM), Ornstein-Uhlenbeck (OU), and white-noise models to continuous color traits using the fitContinuous function of the R package geiger version 2.0.6.4 (Pennell et al. 2014), implemented in phytools. For each trait, we evaluated model support by comparing AIC weights among models. However, given the criticisms of AIC for phylogenetic model selection, we also performed Phylogenetic Monte Carlo (PMC) analyses, implemented in the R package pmc, to assess model support as well as statistical power to discriminate among competing models (Boettiger et al. 2012). PMC analyses were run with 500 bootstrap replicates and by testing all 3 possible pairwise model comparisons.

Ancestral state reconstruction for continuous traits was done with the contMap function of phytools using likelihood estimation (anc.ML), according to their best-fit model of trait evolution. For the dorsal color metric and pterin concentration, this was Brownian motion (Supplementary Table S3; Supplementary Fig. S3). For carotenoid concentration and reflectance λ_max_, this was OU, which was marginally favored over white noise in the PMC analyses (Supplementary Fig. S3).

Ancestral state reconstruction for discrete traits was done with stochastic character mapping (make.simmap function in phytools), an equal rates model (model = ER), and 1000 simulations. We found that the ER model gave much more parsimonious reconstructions (minimizing the number of state changes) than an all-rates-different model, and thus used ER for each trait. The traits used in these analyses were presence/absence of different pigments based on the TLC data. When reporting numbers of state changes, we give the mode (most frequent number of state changes), in addition to the mean and standard errors, which were calculated from the 1000 simulations of the stochastic character mapping.

We calculated phylogenetic signal for continuous traits using Blomberg’s K (Blomberg et al. 2003), implemented in phytools with the phylosig function, with 10,000 simulations. For discrete traits, we used the method of Fritz and Purvis (2010). This method calculates a metric, *D*, which can take negative values when traits are clumped (e.g., a trait evolving once and being retained in all descendants), values around zero when consistent with Brownian motion, and values above 1 when random or overdispersed. We calculated *D* using the phylo.d function of caper version 1.0.1 with *P*-values based on 10,000 permutations (Orme et al. 2013).

## Results

### Phylogeny

Across all 38 samples included in the phylogenetic analysis, we recovered a total of 3258 UCE loci. After sequence trimming and filtering for completeness (keeping only loci present in 95% or more of taxa), the data set was reduced to 1550 UCE loci. Our final alignment consisted of 786,579 characters, of which 57,236 were parsimony-informative. Average missing data per sample was 4.95%. ModelFinder selected 49 partitions, each including from 1 to 80 loci. Partitions and their corresponding models of nucleotide evolution are given in Supplementary Table S4. The optimal phylogenetic tree had a log-likelihood of −2268095.948. We found 26 slightly suboptimal trees (i.e., a difference < 0.1 log-likelihood unit with the optimal tree) but their topologies were identical to the optimal tree (only the branch lengths differed). As expected with our ratio of informative characters to nodes, UFBOOT values are high. In general, concordance factors are medium to high, but with low values associated with some of the more recent splits such as the most exclusive clade including *R. defleri* and *R. summersi*.

Consistent with previous studies (Grant et al. 2006, 2017; Santos et al. 2009; Brown et al. 2011; Muell et al. 2022), our phylogenetic analysis of *Ranitomeya* based on UCEs recovered two major clades, herein labeled the *vanzolinii* clade and the *fantastica* clade (Fig. 1). For an updated infrageneric taxonomy, we refer the reader to Muell et al. (2022). We also note that, based on their phylogenetic position, sampling location, and morphology, it is currently not possible to assign the Catuaba and Envira populations to any known species, and are thus referred to as “sp.” herein. *Ranitomeya cf. yavaricola* bears morphological similarity to bona fide *R. yavaricola,* but as this population is ~300 km S from the type locality, we use the label *cf.* to express uncertainty regarding the species designation.

Despite our inclusion of a single sample per taxon, our phylogenetic results are similar to those of Muell et al. (2022). Most differences are associated with different sampling within species, such as the inclusion of additional sampling localities (e.g., *R. sirensis* and *R. uakarii*). However, the relative position of *R. defleri* and *R. toraro* deserves further comparisons. When the UCE data set was analyzed allowing for different models of evolution for different parts of the data, these two species were nested within the *fantastica* clade and sister to the *reticulata* group sensu Muell et al. (2022) (BS = 100%, Fig. 1, Supplementary Fig. S2). On the other hand, analyses applying a single model of evolution for all the data (Supplementary Fig. S4) or a quartet-based approach (Muell et al. 2022) recovered *R. toraro* as sister of all the other species of the *fantastica* clade (BS = 100%). We present and discuss our results on the basis of the former topology as it represents a more intensive tree search and considers that different parts of the genome can change under different models of nucleotide evolution. However, we also performed all downstream analyses using the topology shown in Supplementary Figure S4 and the main results of our study did not change.

### Dorsal Color Evolution

**Figure 2.**
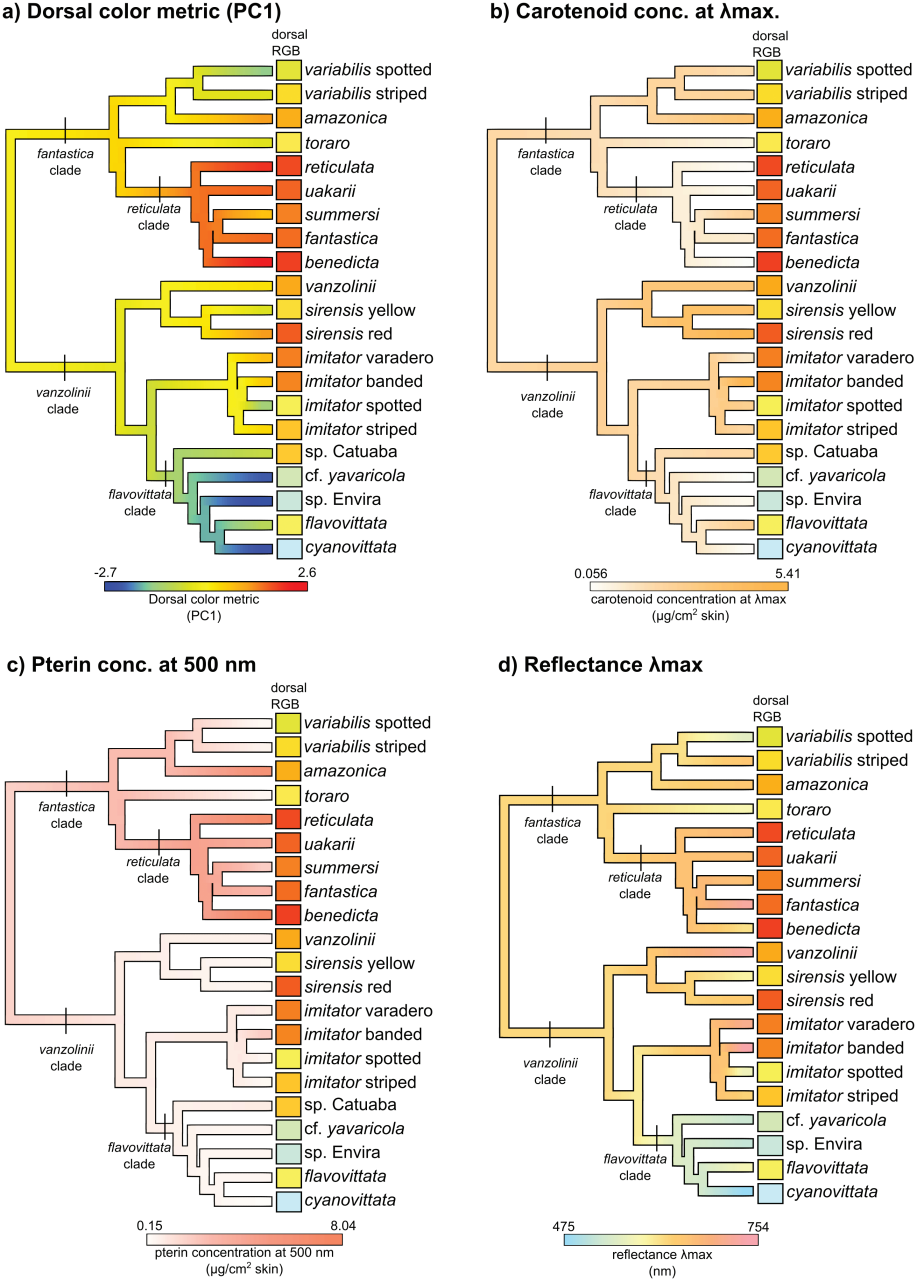
Ancestral state reconstructions for 4 continuous traits. (a) Dorsal color metric PC1 (58.2% of variation), derived from reflectance spectra, (b) Carotenoid concentration, derived from chloroform absorbance spectra, (c) Pterin concentration at 500 nm, derived from ammonia absorbance spectra, and (d) Wavelength of peak reflectance (reflectance λmax), derived from dorsal reflectance spectra. Note that the pterin concentration is based on absorbance at 500 nm and is thus indicative of drosopterin concentration. For b, c, and d, the color scales are representative of the color gamut controlled by that trait; for a, the color scale is a standard blue-to-red color ramp which approximately coincides with the observed colors of these frogs. The rectangles at the tips show the RGB dorsal color of that taxon.

Our PCA of dorsal reflectance spectra yielded two main principal components (eigenvalues > 1). Principal component 1 explained 58.2% of the variation, loading positively on quantum catches for S (short-wavelength cone, loading = 0.549) and M (medium-wavelength cone, loading = 0.517), and negatively on L (long-wavelength cone, loading = −0.653). Biologically, this means that most color variation involves correlated changes in the short to medium wavelength range, with opposite changes in the long wavelength range (e.g., blue frogs with high reflectance in the blue-green range and low in the red; or red frogs with high reflectance in the red range and low in the blue-green). The second principal component explained 27.6% of the variation, loading heavily on U (ultraviolet cone, loading 0.908). For further analysis, we analyzed only PC1, which we refer to as the dorsal color metric (Fig. 2a).

More generally, most taxa within *Ranitomeya* were found to have yellowish-orange dorsal coloration; our analyses further indicate that yellowish-orange dorsal coloration was the ancestral state both for *Ranitomeya* overall as well as the *fantastica* and *vanzolinii* clades (Figs 1 and 2a). In this context, color transitions and convergence can be more clearly defined as they generally involve the transition from yellowish-orange to some other color. Bluish-green dorsal coloration evolved once within *Ranitomeya* in the *flavovittata* clade (Fig. 1). Within this clade, *R. flavovittata* regained yellow coloration from a blue/green ancestral state. Other gains of yellow or greenish coloration (e.g., the spotted morphs of *R. variabilis* and *R. imitator* and the yellow morph of *R. sirensis*) involve a color shift in the opposite direction, that is, yellow or greenish coloration evolving from an orange ancestral state.

Our analyses reveal at least two independent gains of red coloration: once in the *reticulata* clade, and once in the red morph of *R. sirensis.* Other gains of reddish coloration are more subtle in that the species did not evolve red coloration per se, but rather evolved redder coloration compared to the inferred ancestral state. For example, in *R. amazonica* and the banded and varadero morphs of *R. imitator*, each has a deeper reddish-orange coloration compared to their sister taxa as well as their ancestral state (Fig. 1).

### Carotenoids

Chloroform skin extracts, which contain carotenoid pigments, were generally yellow across most samples. Absorbance spectrometry of these extracts confirmed the presence of carotenoids, which usually have characteristic triple-peak absorbance spectra and absorbance maxima typically in the range of 450–460 nm in the case of chloroform extracts (Supplementary Fig. S1). Carotenoids were further confirmed with TLC (see below).

Although most samples contained carotenoids based on the chloroform absorbance spectra, there were some notable exceptions. Within the *vanzolinii* clade, carotenoid reduction or loss was observed in taxa with blue or bluish-green coloration, that is, *Ranitomeya cyanovittata, R.* cf. *yavaricola,* and *R.* sp. Envira (Fig. 2b, Supplementary Fig. S5). In the *fantastica* clade, carotenoid loss or reduction was found in taxa with red coloration (Fig. 2b, Supplementary Fig. S5). *Ranitomeya benedicta*, which has vivid red coloration on the head, had no detectable carotenoids from the chloroform absorbance spectra (although trace beta-carotene was found with TLC; see below), while *R. reticulata* and *R. uakarii,* which are also red, had very low amounts of carotenoids (Supplementary Fig. S5). By contrast, the red morph of *R. sirensis*, which is the only red taxon in the *vanzolinii* clade, had a distinctly red chloroform extract due to its high carotenoid concentrations, including red C-4 ketocarotenoids (Twomey et al. 2020a).

Ancestral state reconstruction of carotenoid concentration (Fig. 2b) suggests that the ancestral state in *Ranitomeya* was an intermediate carotenoid concentration, with two subsequent reductions: once in the *reticulata* clade and once in the *flavovittata* clade. Carotenoid concentrations showed independent increases from a lower ancestral state in *R. summersi, R. flavovittata*, the banded morph of *R. imitator,* and, to a lesser extent, in the most recent common ancestor of *R. vanzolinii* and *R. sirensis.*

**Figure 3.**
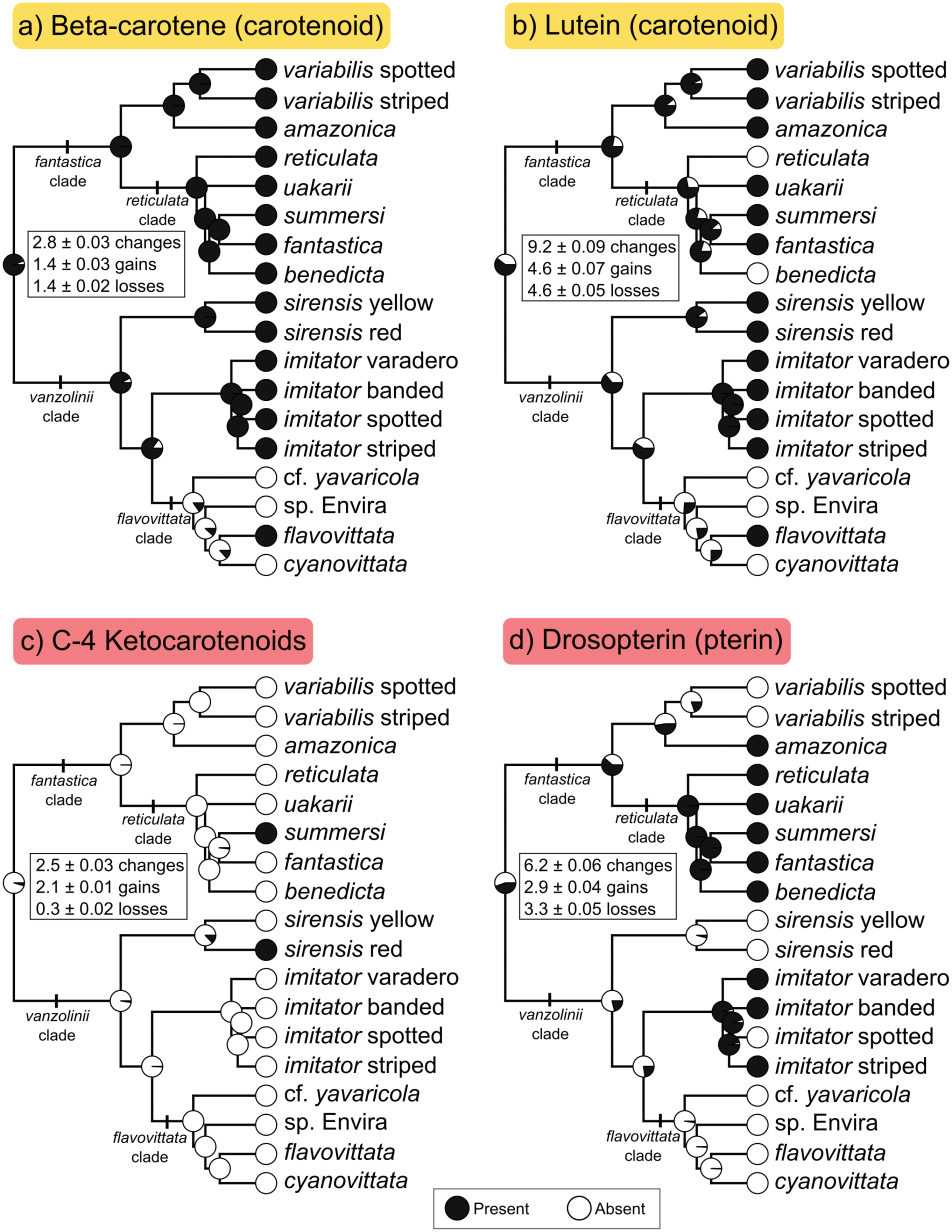
Ancestral state reconstructions for presence/absence of carotenoid and pterin pigments in Ranitomeya. (A) Beta carotene (carotenoid), (B) Lutein (carotenoid), (C) C-4 ketocarotenoids (carotenoid), and (D) Drosopterin (pterin). Pigments were scored as present/absent based on TLC results and absorbance spectra. Pigment color (red/yellow) is indicated with the label color. Observed states are shown at the tips; pie charts at internal nodes represent posterior probabilities of each state. Boxes report the mean ± standard error for the numbers of total changes, gains, and losses calculated from the 1000 stochastic mapping iterations. For 3 taxa (*R.* cf. y*avaricola*, *R.* sp. Envira, and *R. cyanovittata*), pigments were scored as absent based on absorbance spectra that indicated a lack of carotenoids and drosopterin. Other species that had skin carotenoids but for which we did not have TLC data (*R. vanzolinii*, *R. toraro*, and *R.* sp. Catuaba) were omitted from this analysis. Results from 3 additional pigments are shown on Supplementary Figure S6.

Using TLC, we were able to identify a number of the skin carotenoids. The most widespread were the dietary carotenoids beta-carotene and lutein (Fig. 3). Beta-carotene was present in all taxa except for *R.* cf. *yavaricola, R. cyanovittata*, and *R.* sp. Envira, and stochastic character mapping indicated presence at the root (posterior probability = 0.94), followed by a single loss (mean = 1.4 ± 0.03) and single gain (mean = 1.4 ± 0.03). Lutein was also absent in these 3 taxa, as well as *R. benedicta* and *R. reticulata*, and most likely present at the root (posterior probability = 0.63), followed by 4 losses (mean = 4.6 ± 0.05) and 4 gains (mean = 4.6 ± 0.07), making it the most evolutionarily labile skin pigment (Fig. 3). Red C-4 ketocarotenoids have been found previously in *R. sirensis* and *R. summersi* (Twomey et al. 2020a, 2020b). We did not find C-4 ketocarotenoids in any other sample and thus their presence remains restricted to these two species. Character mapping indicates absence at the root (posterior probability = 0.97), with two independent gains (mean = 2.1 ± 0.01) in *R. summersi* and the red morph of *R. sirensis,* and no losses (mean = 0.3 ± 0.02). Results from other carotenoids are given in Supplementary Fig. S4. Phylogenetic signal of categorical traits (*D*) was negative for beta-carotene (*D* = −0.54), canary xanthophyll (*D* = −1.4), and drosopterin (*D* = −0.14), consistent with “clumped” trait distributions where traits evolve few times and are retained in descendants; similarly, *P*-values for these 3 pigments rejected the possibility of random trait evolution. In the other pigments, *D* was positive and consistent with a random phylogenetic pattern.

### Pterins

Ammonia-based skin extracts, which contain pterin pigments, were mostly colorless or pale yellow in species of the *vanzolinii* clade (Fig. 1). However, all the species in the *fantastica* clade that had orange or red dorsal coloration also had orange/red pterin extracts (Fig. 1) with absorbance peaks or shoulders around 500 nm (Supplementary Fig. S1b), characteristic of the pterin pigment drosopterin. TLC of pterin extracts confirmed the presence of large quantities of drosopterin in these species, in particular *R. reticulata* and *R. benedicta* (Supplementary Fig. S5). In the *vanzolinii* clade, drosopterin is known from *R. imitator* (Twomey et al. 2020b), and we did not detect it in any of the other species in this clade. Stochastic character mapping of drosopterin reveals a slightly higher probability of absence at the root (posterior probability 0.58), with 3 gains (mean = 2.9 ± 0.04) and 3 losses (mean = 3.3 ± 0.05); notable putative gains involve at the base of the *reticulata* clade, and in the species *R. imitator* and *R. amazonica* (Fig. 3). For results on other pterin pigments, see Supplementary Material.

### Reflectance Spectra and Structural Coloration

All dorsal reflectance spectra, except for *Ranitomeya* sp. Catuaba, had a hump-shaped reflectance curve (Fig. 1) that is suggestive of a color contribution from structurally colored iridophores. Coherently scattering iridophores are predicted to yield similar hump-shaped reflectance curves where the location of the spectral peak is controlled in large part by the thickness of the iridophore platelets (Morrison 1995; Saenko et al. 2013). Reflectance curves based on purely structural coloration are expected to be roughly symmetrical around the peak, although here we observed that reflectance spectra typically have a steeper slope left of the peak, likely due to carotenoid and pterin pigments absorbing these shorter wavelengths. In some taxa, such as *R. cf. yavaricola*, *R. sp.* Envira, and *R. cyanovittata,* which largely lack carotenoid and pterin pigments (Supplementary Fig. S1), structurally colored iridophores represent the most plausible mechanism generating the observed colors. Similarly, *R. toraro* was found to have a relatively small color contribution from carotenoid and pterin pigments, yet this species has a bright yellow dorsal coloration and peaked reflectance spectrum (Fig. 1); Twomey et al. (2020b) also reported that some individuals of *R. imitator* also had bright yellow or orange dorsal coloration despite low concentrations of carotenoid and pterin pigments.

*Ranitomeya* sp. Catuaba is the lone species whose dorsal reflectance spectrum is not distinctly hump-shaped, instead sloping up continuously past the edge of the measurement range, unlike any of the other species (Fig. 1). This raises the possibility that structural coloration, if any, is through some other mechanism (e.g., light reflection from underlying integument) and not due to iridophores. For this reason, this species was removed from our comparative analysis of reflectance λ_max_ (Fig. 2d).

Across *Ranitomeya*, there is a general relationship between reflectance λ_max_ and dorsal coloration, with orange and red frogs typically having peaks > 650 nm, yellow frogs approximately 580–650 nm, and blue or blue-green frogs 475–527 nm. This relationship is not unexpected given that it is taken directly from reflectance spectra and is therefore a proxy measure of color. Still, there are some exceptions to this pattern. *Ranitomeya benedicta* has bright red coloration on the head, but a strongly peaked reflectance spectrum located at 620 nm, suggesting that the underlying structural coloration is in fact yellow. *Ranitomeya vanzolinii* has a reflectance spectrum that peaks outside the visible spectrum at 740 nm, suggesting that the yellowish-orange coloration of this species is due to red-shifted structural coloration combined with high concentrations of yellow carotenoids.

The ancestral state reconstruction of our proxy for structural color, reflectance λ_max_, suggests ancestral orange-yellow structural coloration for *Ranitomeya*. Subsequently, there were at least 3 independent shifts toward shorter wavelengths (once in the *flavovittata* clade, and once each in the spotted morphs of *R. imitator* and *R. variabilis*), and at least 4 independent shifts toward longer wavelengths (*R. fantastica*, *R. flavovittata*, banded *R. imitator*, and *R. vanzolinii*).

### Evolutionary Models of Trait Evolution

For the dorsal color metric (Fig. 2a) and pterin concentration (Fig. 2c), we found significant phylogenetic signal (dorsal color metric: *K* = 0.762, *P* = 0.002; pterins: *K* = 0.719, *P* = 0.014). For both traits, best-supported model of trait evolution was Brownian motion (BM), while white noise was strongly rejected in both cases (Supplementary Fig. S3, Supplementary Table S3). For carotenoid concentration and reflectance λ_max_, phylogenetic signal was not statistically significant (carotenoids: *K* = 0.411, *P* = 0.361; reflectance λ_max_: *K* = 0.415, *P =* 0.271). According to the phylogenetic Monte Carlo (PMC) analysis, OU was marginally better supported than white noise for both traits, while AICc ranked white noise slightly higher. However, OU was also considered plausible for both traits (ΔAIC_c_ ≈ 2, Supplementary Table S3).

Interestingly, our most direct measure of the color phenotype, the dorsal color metric, had the strongest phylogenetic signal, indicating that closely related species in *Ranitomeya* are more likely to have similar dorsal colors than distantly related species. A similar pattern was found with respect to pterin concentration at 500 nm (indicative of drosopterin content), likely driven by high quantities of this pigment in several closely related species in the *reticulata* clade (Supplementary Fig. S5). However, in two other mechanistic traits (carotenoid concentration and reflectance λ_max_), this relationship with the phylogeny is much weaker. Overall, this suggests that although the color phenotype itself has a fairly strong relationship with the phylogeny, 2 of the 3 mechanistic traits do not. We see this as a function of redundancy among mechanisms, where specific color phenotypes can be produced by various mechanistic combinations, and in some cases these mechanisms may be negatively correlated (e.g., production of red color through distinct pigmentary routes; see below).

## Discussion

**Figure 4.**
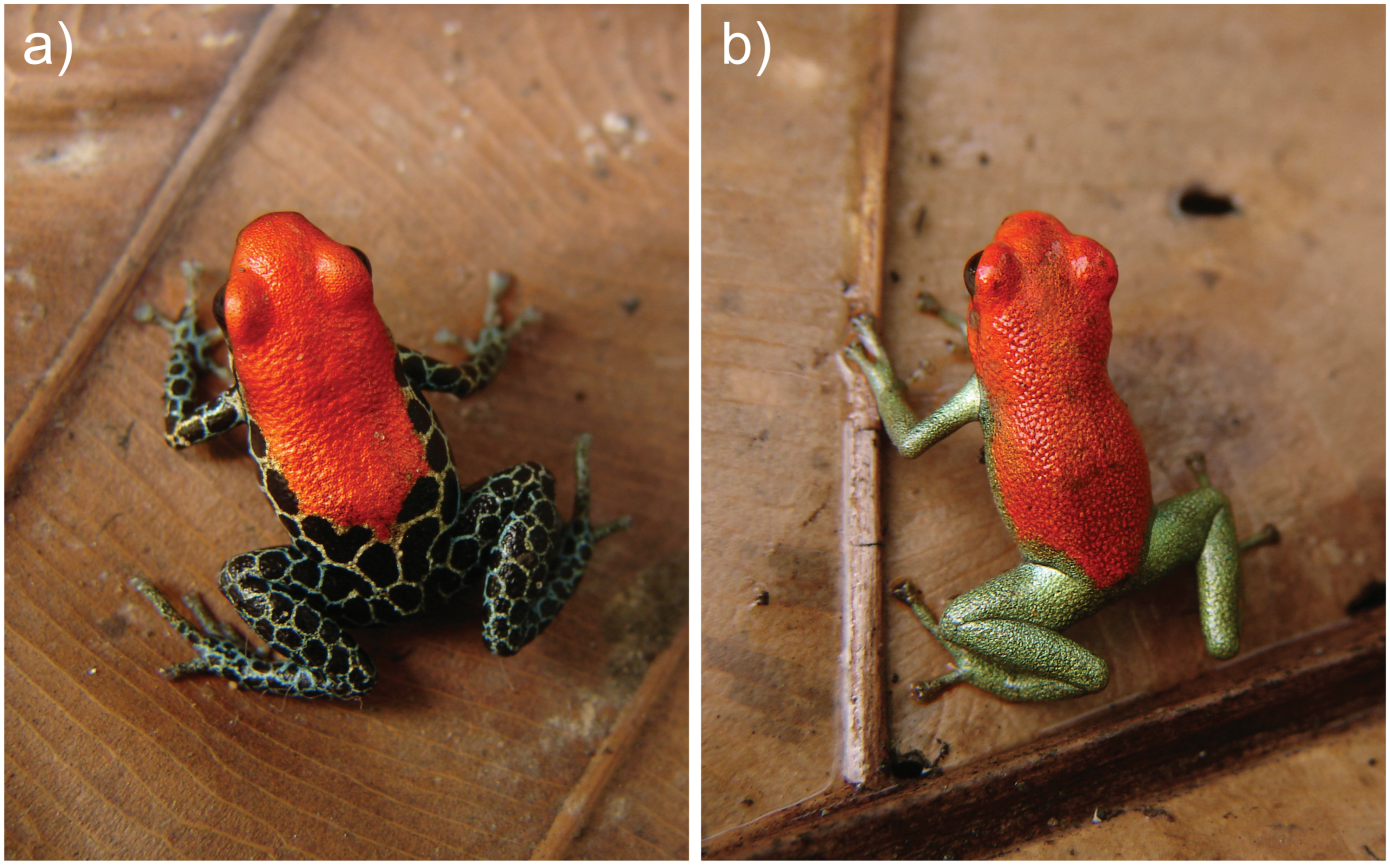
Convergent evolution of red coloration via different mechanisms in *Ranitomeya*. (a) Drosopterin-based red coloration in *R. reticulata,* Iquitos, Peru. (b) Ketocarotenoid-based red coloration in *R. sirensis*, Cordillera El Sira, Peru.

The goal of this study was to investigate whether color convergence within a clade of closely related species occurs through the same (parallel) or different (nonparallel) physical mechanisms. Because convergent evolution requires that phenotypes evolve independently in each lineage, identifying convergent phenotypes, particularly among close relatives, requires an explicit phylogenetic hypothesis. By examining color evolution in an explicit phylogenetic framework, we found several cases of color convergence. Further, by reconstructing the evolution of specific color mechanisms, we found that both parallel and nonparallel mechanisms explain this convergence. The clearest example of nonparallel convergence is the pterin- and carotenoid-based red pigmentation in the *reticulata* clade and *R. sirensis* (Fig. 4). In the former, the reddish coloration of all 5 species is largely attributable to an increase in the red pterin pigment drosopterin (Fig. 2c, Supplementary Fig. S1b). Most notable of these species are *R. benedicta* and *R. reticulata*, which have the highest concentrations of drosopterin in the genus and almost entirely lack carotenoid pigments (Supplementary Fig. S5). In contrast, *R. sirensis* lacks drosopterin but has high concentrations of red ketocarotenoids. Ketocarotenoids and drosopterin are functionally similar in that they are both red pigments with similar absorbance spectra (e.g., hump-shaped with strong absorbance at 480–500 nm), but they originate from unrelated pathways. Other cases of color convergence can be best understood as parallel evolution, particularly with respect to structural coloration. Previously, it was shown that the hue of the dorsal color in *Ranitomeya* was strongly correlated to the thickness of iridophore platelets, indicating that quantitative variation in a single trait can explain a large proportion of color variation in these frogs (Twomey et al. 2020b).

These observations paint a complex picture where coloration evolves through coordinated changes in a suite of mechanisms and some cases of color convergence are not easily classified as parallel or nonparallel. This can be understood by considering the dermal chromatophore unit of amphibians as a complex phenotype, where different components (e.g., cell types and pigments contained therein) may be products of different pathways with different evolutionary histories. Xanthophores, which are the superficial pigment-containing cells, are especially complex in that they have various types of subcellular vesicles that contain suites of carotenoid and pterin pigments. A good example of this mechanistic complexity is in *Ranitomeya summersi*, where the orange coloration is a product of 1) drosopterin, a conserved pigment within this clade, 2) ketocarotenoids, derived independently in this species (Fig. 3), and 3) orange-tuned structural coloration, which is “conserved” in the sense that iridophore platelet variation is a common axis of color variation across most species of *Ranitomeya.*

Whereas convergence through parallel mechanisms indicates a prominent role of homology in shaping phenotypic evolution, nonparallel convergence implies that new phenotypes can and do evolve through new solutions. However, this distinction of mechanisms as “same” or “different” is not always straightforward and could mean same pathway, same gene, or same nucleotide, depending on the level of focus of a particular study (Manceau et al. 2010; Elmer and Meyer 2011; Pearce 2012; Rosenblum et al. 2014). An oft-cited example of different mechanisms leading to convergent phenotypes is in the recurrent evolution of white phenotypes through the loss of melanin (Arendt and Reznick 2008). In particular, the result that mutations in the same or different genes have led to repeated evolution of white coloration demonstrates that there are many ways to disrupt the melanin pathway to yield a convergent white phenotype (Hoekstra et al. 2006; Rosenblum et al. 2010). However, these examples also underscore the fact that white coloration essentially involves a loss-of-function mutation somewhere within the highly conserved melanin pathway. An important insight, as emphasized by Pearce (2012) and Leander (2008) is that, although the specific mutations that caused the convergent phenotype evolved independently and are thus not homologous, they all affect the same conserved pathway. This demonstrates the importance of internal forces in phenotypic convergence and suggests that the role of homologous pathways, at least in the case of melanin-based color convergence, is high. It also suggests that loss-of-function phenotypes (e.g., abrogation of melanin production or, as described below, carotenoid accumulation) may be more likely to represent parallel convergence as there are many ways to break a particular pathway (Rosenblum et al. 2010).

A key question thus becomes whether independent gains of the same pigments occurred through the same changes within existing pathways. With drosopterin, our results indicate 2–3 independent gains of this pigment within *Ranitomeya* (Fig. 3). It is likely that these are products of the activation of a conserved pathway given that much of the pterin synthesis pathway is highly conserved (Andrade and Carneiro 2021). Furthermore, in *Ranitomeya* species lacking drosopterin, other colorless pterins such as isoxanthopterin and pterin are observed (Twomey et al. 2020b), both of which are derived from drosopterin’s precursor molecule. This suggests that even in species that lack drosopterin, much of the synthesis pathway remains intact and that drosopterin quantity is likely regulated by subtle alterations to the ratios or quantities of key precursor molecules.

With respect to red carotenoid pigmentation, the degree of pathway similarity among species is unclear, as red carotenoids can be either sequestered directly from the diet or metabolically converted from yellow dietary carotenoids (McGraw 2006; Toomey et al. 2022). With metabolic conversion, a ketolase enzyme essentially builds upon the existing carotenoid pathway to add a novel function, although different animal groups appear to use different, but related, enzymes. For example, birds (and some non-avian reptiles) appear to have a conserved carotenoid ketolase (CYP2J19) belonging to the cytochrome P450 (CYP) class (Lopes et al. 2016; Twyman et al. 2016, 2018), while *Ranitomeya sirensis* appears to have a different CYP enzyme, CYP3A80 (Twomey et al. 2020a). At first glance, this would imply that homology is of less relevance in the recurrent evolution of ketocarotenoid coloration, as different enzymes were independently recruited for the same function across different animal groups. On the other hand, all known or suspected carotenoid ketolases in animals are CYP enzymes (Mundy et al. 2016; Wybouw et al. 2019; Twomey et al. 2020a; Huang et al. 2021). Many CYPs mediate xenobiotic elimination through the addition of ketone groups to diverse substrates, including several carotenoid-like molecules (Cooper et al. 2011). In this sense, the evolution of CYPs for xenobiotic removal may be thought of as a “potentiating mechanism” for carotenoid ketolase function.

## Conclusions

The study of phenotypic convergence continues to shed light on fundamental processes of evolution, such as understanding the relative importance of internal and external forces and whether evolutionary outcomes are deterministic or contingent on specific details (Blount et al. 2018). As study systems are identified and investigated, understanding convergence at deeper levels of hierarchical organization becomes possible. Currently, our understanding of convergence at the molecular genetic levels, such as the genes, alleles, and nucleotides resulting in color convergence, is limited by our lack of detailed knowledge on vertebrate coloration pathways. In particular, the genes and pathways that regulate pterin pigmentation and structural coloration remain poorly understood, although this is changing (Andrade et al. 2019; Stuckert et al. 2019, 2021; Rodriguez et al. 2020). Ultimately, this needs to be integrated with a better understanding of the signaling functions and possible constraints on pigment expression (Koch and Hill 2018; Hill et al. 2019; Stuart-Fox et al. 2021b). This could clarify why mechanistic redundancy exists and why color signals evolve through different evolutionary pathways across species, exemplified here by the alternate routes to red coloration in *R. sirensis* and *R. reticulata* (Fig. 4). Here, we have focused on identifying color convergence in a young radiation of poison frogs, and studying the basis of this convergence at the most proximate level. Our results demonstrate that similar coloration evolved through both parallel and nonparallel pathways. This indicates that although homologous mechanisms often provide a shared basis for color convergence, convergence may also proceed through novel mechanisms built on top of preexisting pathways, for example, the independent evolution of red carotenoid pigmentation. Our study also uncovered an exciting, and considerably more complicated, array of mechanistic building blocks, which suggest that color evolution, and its repeated convergence, can occur through both “same” and “different” mechanisms even among closely related species.

## Supplementary Material

Data available from the Dryad Digital Repository: http://dx.doi.org/10.5061/dryad.gf1vhhmsn

## Data Availability

The data underlying this article are available on Dryad under the DOI:10.5061/dryad.gf1vhhmsn. Pigment data from previous publications is available at https://doi.org/10.5061/dryad.z34tmpg8f and https://doi.org/10.5061/dryad.wpzgmsbj2 Sequencing reads for the phylogenomic analysis are available on the European Nucleotide Archive under the project accession PRJEB64543; accession numbers of individual samples are given in Supplementary Table S2.
